# Fibronectin: Molecular Structure, Fibrillar Structure and Mechanochemical Signaling

**DOI:** 10.3390/cells10092443

**Published:** 2021-09-16

**Authors:** Caleb J. Dalton, Christopher A. Lemmon

**Affiliations:** Department of Biomedical Engineering, Virginia Commonwealth University, 401 W. Main St., Richmond, VA 23284, USA; cjdalton@vcu.edu

**Keywords:** fibronectin, fibrillogenesis, extracellular matrix, biomechanics, mechanobiology

## Abstract

The extracellular matrix (ECM) plays a key role as both structural scaffold and regulator of cell signal transduction in tissues. In times of ECM assembly and turnover, cells upregulate assembly of the ECM protein, fibronectin (FN). FN is assembled by cells into viscoelastic fibrils that can bind upward of 40 distinct growth factors and cytokines. These fibrils play a key role in assembling a provisional ECM during embryonic development and wound healing. Fibril assembly is also often upregulated during disease states, including cancer and fibrotic diseases. FN fibrils have unique mechanical properties, which allow them to alter mechanotransduction signals sensed and relayed by cells. Binding of soluble growth factors to FN fibrils alters signal transduction from these proteins, while binding of other ECM proteins, including collagens, elastins, and proteoglycans, to FN fibrils facilitates the maturation and tissue specificity of the ECM. In this review, we will discuss the assembly of FN fibrils from individual FN molecules; the composition, structure, and mechanics of FN fibrils; the interaction of FN fibrils with other ECM proteins and growth factors; the role of FN in transmitting mechanobiology signaling events; and approaches for studying the mechanics of FN fibrils.

## 1. Introduction

The extracellular matrix (ECM) is a substrate for cells that modulates migration, proliferation, differentiation, spreading and survival by serving as both a molecular reservoir and a structural scaffold with tissue-specific mechanical properties. A web woven of secreted fibrillar proteins, its protein composition is also specific to tissue type, though two major structural themes have been identified: a thread-like interstitial network that is present between and around cells; and a pericellular sheet-like basement membrane that serves as a cellular platform and a boundary around cells [1,2,3]. Despite their structural differences, fibrillar interstitial matrices and pericellular basement membranes share similarities in their initial assembly and overall composition and are constructed of four major protein classes: collagens, elastins, proteoglycans (PGs), and glycoproteins [1,2].

Collagens, elastins, and PGs play a primarily structural role in the ECM. These proteins are either synthesized as precursor elements (procollagen, tropoelastin) and crosslinked into their fibrillar structures by lysyl oxidase (LOX) or consist of peptide units covalently linked to carbohydrates that aggregate into enmeshed networks [4,5,6]. Their assembly is mediated via cell attachment/stretch and/or protein self-association, resulting in structures with unique mechanical properties [1,7,8,9]. Collagen cross-linking density and post-translational modifications provide tensile strength and structural integrity; elastin hydration and flexibility are stretch-state dependent, giving tissues their elasticity and resilience; and PGs bind water by coulombic attraction, providing stiffness and compressive resistance.

In contrast, glycoproteins contain few, if any, repeating structures and primarily serve as connectors within the ECM [1]. Arguably, it is the presence of these glycoprotein linkages that enrich and stabilize the ECM as they mediate cell–ECM, ECM–ECM and ECM-soluble factor connectivity. There are two predominate glycoproteins found in the ECM: laminin, exclusively restricted to basement membranes, and fibronectin (FN), abundantly found in highly organized structures in both interstitial and basement membrane ECMs [2,10,11,12]. Although the initiation of basement membrane assembly does not involve FN, all primordial ECM assembly does involve FN matrix assembly. Since FN fibrils are one of the earliest ECM proteins assembled in tissue development and wound healing, and because FN molecules contain multiple domains that bind several ECM proteins, growth factors, and small molecules, it has become evident that the understanding of assembly, molecular storage, and cellular interaction within the ECM is dependent on the understanding of FN fibril assembly and its interaction with cells, other ECM proteins, and soluble signaling proteins [1,13].

## 2. Fibronectin: The Molecule

**Figure 1:**
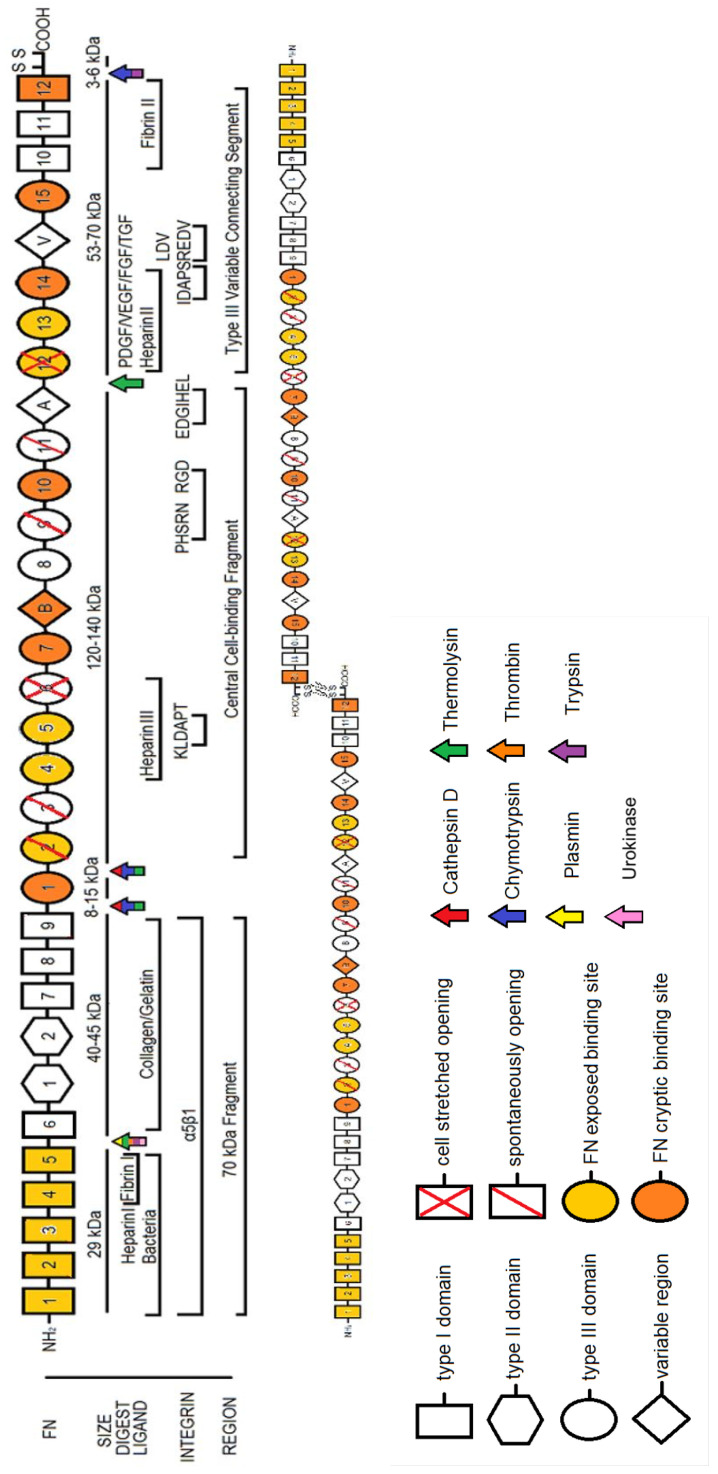
A schematic of the domains of FN with relevant structural features, cleavage sites, nomenclature, and integrin binding. FN is an approximately 250 kDa protein that is secreted as a dimer. Individual domains are classified as Type I (rectangles), Type II (hexagons), Type III (ovals), or a variable region (diamond). Domains that spontaneously open are shown with a single red slash, while domains that are mechanically unfolded are shown with a red X. Domains that have exposed FN–FN binding sites are shown in gold, while FN domains that have been shown to exhibit cryptic FN–FN binding sites are shown in orange. Molecular weight of regions are directly below. Enzymes known to digest FN are shown at their specific sites with arrows and color coded appropriately. Regions and/or specific sequences that have been shown to bind other ECM constituents are labeled based on size and ligand, then integrins are listed below that, and common terminology for each FN fragment is listed below that. The dimerization of FN at its C-terminus is shown at bottom.

FN is a glycoprotein whose size ranges from 230 to 270 kDa and usually exists as a dimer, covalently linked by a pair of disulfide bonds at the C-termini (Figure 1) (see [14] for a review of early work on FN). Each monomer consists of three repeating units: 12 Type I, 2 Type II, and 15–17 Type III domains which combined account for 90% of the FN sequence. Structural homologs of the Type I, II and III domains exist in other biomolecules, suggesting that FN evolved through exon shuffling [1,13]. Despite originating from a single gene, as many as 20 different human variant and 12 different rodent and cow variant FN isoforms have been identified, indicating alternative splicing mechanisms unique to species and tissue development. Exon usage or skipping gives rise to the presence of two distinct Type III repeats, EDA (‘EIIIA’, ‘EDI’) and EDB (also ‘EIIIB’ or ‘EDII’), each coded by a single exon. Similarly, a variable length V region (‘IIICS’) can be included or excluded in the Type III connecting segment.

Conversely, individual FN domains themselves are relatively consistent from molecule to molecule. NMR structures of Type I domain pairs show conservation in their tertiary structure. Their modules connect end to end while twisting clockwise about the long axis whose relative orientation is fixed by hydrophobic contacts [15]. Only I${}^{8}$ is known to break this conformation, by forming, along with I${}^{7}$ and I${}^{9}$, a β-sheet super domain [16]. Type II domains have large buried surface areas but seem to require paired configurations for functionality, as FN suffers near-complete loss of gelatin-binding activity when II${}^{1}$ and II${}^{2}$ are separated. Furthermore, their compact, globular structures are arranged in a hairpin configuration, suggesting that this region of FN is not simply a ‘string of beads’ structure [17]. Type III domains, on the other hand, extend out in rod-like structures composed of seven-stranded ‘β-sandwiches’ and, unlike Type I and Type II domains, contain no internal disulfide bonds. As such, they are not crosslinked and may undergo conformational changes, revealing buried or ‘cryptic’ binding sites for various ECM constituents [18,19]. Steered molecular dynamics simulations suggest that, before unfolding, these domains unravel, twisting to align β-strands before rupturing open [20]. Opening of domains III${}^{6}$ and III${}^{12}$ via cell-mediated forces has been identified experimentally using thiol reactive dyes that probe for buried cysteine residues [21]. Interestingly, other domains, including III${}^{2,3,9,11}$, transiently unfold in the absence of applied force [22,23,24]. Domains that open via force or spontaneously are indicated in Figure 1. While all Type III domains in FN have a homologous structure, studies have shown that each Type III domain in FN varies widely in terms of mechanical and chemical stability [13,25], which gives rise to a protein in which different domains will unfold under different forces relative to their neighbors. These distinct characteristics of both domain type and domain number, together with their relative positioning and FN’s overall tertiary configuration, give rise to FN’s unique attributes as a biomolecule in the ECM.

### 2.1. Plasma FN vs. Cellular FN

FN is subcategorized as either plasma FN (pFN), predominantly synthesized by liver hepatocytes, or cellular FN (cFN), which is produced by a wide variety of cells including fibroblasts, chondrocytes, myocytes, and synovial cells. pFN circulates in the blood at a high concentration (approximately 300 µg/mL), while cFN is locally secreted. pFN and cFN both regulate cell attachment and spreading, although the two types exhibit differences in solubility, binding, size, and proteolytically generated fragments [26]. cFN has been shown to be 50 times more potent in restoring normal fibroblast alignment and morphology after cell transformation [26] and also displays 150 times more effectiveness in hemagglutination than pFN. Previous studies have also suggested differences in pFN-derived FN fibrils, which form shorter fibrils, from cFN-derived fibrils, which are more extended. This is potentially the result of differential binding of transglutaminases, which crosslink FN fibrils [27]. pFN and cFN are bound by distinct transglutaminases [28] and also exhibit differential cross-linking by transglutaminase: cFN forms a very high molecular weight complex but does not form the intermediate multimers observed in pFN cross-linking [27].

### 2.2. The Role of Alternatively Spliced FN

FN isoforms impact signaling pathways differently due to alternative splicing of domains, and FN molecular variation facilitates tissue-specific FN functions [29]. As discussed above, the primary differences in variable splicing include the presence or absence of the EDA domain, the EDB domain, and the IIICS region. EDA and EDB domains are almost exclusively spliced out from pFN, 50% of which also lack IIICS, whereas cFN exhibits greater heterogeneity, containing none, one or both EDA and EDB regions. EDA and EDB themselves are highly conserved, having nearly identical sequences in virtually all mammals compared to other Type III FN domains that diverge beyond the primate order [13,19,30].

EDA-containing FN isoforms (EDA+ FN) play roles in migration, differentiation, signaling, adult wound healing and overall tissue health, with contesting beliefs on its role in fetal development [29,31]. EDA+ FN has increased binding affinity for integrins α${}_{4}$β${}_{1}$, α${}_{9}$β${}_{1}$, possibly α${}_{4}$β${}_{7}$, and RGD-associated α${}_{5}$β${}_{1}$, which enhances cell motility, proliferation and transformation in osteoblasts and fibroblasts [29,32]. Recombinantly expressed EDA domain associates with Toll-like receptor 4 (TLR4) to generate inflammatory responses in the innate immune system (amnion cells); increased metalloproteinase (MMP) activity, cyclooxygenase 2 (COX2) expression, and prostaglandin E2 expression via the NF-ΚB and ERK1/2 pathways; and cytokine activity through the p38 and MK-2 pathways [31]. Murine studies have shown differential expression of EDA+ FN across injuries, from the wound site through the surrounding muscle into the dermis [33]. Additionally, aberrant healing occurs without the EDA domain, and altered life spans in both knockout and constitutively expressed EDA+ FN show that regulation of FN splicing dynamics may be vital in tissue regeneration and the aging process [33]. EDA has not been identified as a key regulator of embryonic development: EDA in bovine models is expressed minutely in the oocyte, in vitro 2-cell embryos, cumulus cells, lungs, ovaries, uterus, in vitro morula, spleen, ovaries, udder, and cumulus cells; moderately in the placenta; and highly in the (un)hatched blastocyst [34].

EDB-containing FN isoforms (EDB+ FN) play roles in protein stability, vascularization, proliferation, and opsonization (phagocytosis), and has been hypothesized to play a role in inflammation, cell attachment, and pregnancy [19,29,32]. Inclusion of EDB increases the proteolytic sensitivity of FN, suggesting that EDB acts to increase the rate of ECM turnover [35]. Presence of EDB upregulates VEGF expression, angiogenesis, and endothelial proliferation. Interestingly, EDB+ FN may be up to 80-fold higher in cerebrospinal fluid infected with bacterial meningitis, but rather unexplored is its interaction with the inflamed dermis. The EDB domain binds to the vitronectin receptor, α${}_{m}athrmv$β${}_{3}$, facilitating phagocytosis in immune cells. It has shown to shift the RGD binding preference to β${}_{3}$ integrins in osteoblasts, yielding higher differentiation and mineralization. There is increased expression of EDB in the placenta, uterus, cumulus cells, and (un)hatched blastocyst; two isoforms of EDB+ FN are present in the hatched blastocysts, such that FN may mediate multiple effects within development [34].

V-containing FN isoforms (V+ FN) play roles in dimer secretion, solubility, cell adhesion, fibrillogenesis, ligand binding, and coagulation [19]. The V region, unlike EDA and EDB, has extensive splicing events: the exon that encodes the entire V region has at least 5 splice variants in humans (V0, V64, V89, V95 and V120, corresponding to their amino acid length), and also contains the first half of the III${}^{15}$ module. All mammals possess V0, V95 and V120 variants of the V region, while chicken and frogs only have two splice variants, and dog cartilage contains an FN isoform that completely lacks the V region along with III${}^{15}$ and I${}^{10}$. V+ FN exists as homodimers, heterodimers and in monomeric forms; most pFN is a heterodimer of V0 and V+ subunits, whereas cFN almost entirely excludes V0 subunits [13,19]. Importantly, V0-V0 dimers are retained in the ER and degraded intracellularly. This suggests that the V region may serve as a partition for FN between the external cellular environment, body fluids, and tissue matrices. Just as EDA and EDB flank the primary α${}_{5}$β${}_{1}$ integrin binding RGD sequence, EDA and V straddle the heparin II domain. V0-V+ dimers are more efficiently incorporated into fibrin clots than FN homodimers of V+ subunits, which may explain the composition of pFN and thereby, any presence of V0 FN in tissues from interstitial fluid exchange [19]. V may be further refined as sequential segments referred to as IIICS-A, -B, -C. Within the IIICS region, peptides CS1 and CS5 have been identified [36], both of which are recognized by α${}_{4}$β${}_{1}$ and α${}_{4}$β${}_{7}$ integrins [37,38,39,40]. Minimal active amino acid sequences have been identified for each of these sites: LDV for CS1, and REDV for CS5. Additionally, different splice variants of IIICS have demonstrated that the initial sequence of the V region is necessary to conserve heparin binding domain activity that inhibits FN fibril formation and cell spreading; conversely, IIICS-B itself displays a novel GAG-binding site for heparin that stabilizes FN matrix assembly and promotes cell contractility through syndecan-4 binding [30,40,41]. The V variant appears in synovial joints, intervertebral discs, pFN, and certain fetal tissues, including (in bovine models) minutely in the spleen, oocyte, muscles, kidney, morula, uterus, skin; moderately in the liver, and highly in the placenta, cumulus cells, lungs, and blastocyst [34].

### 2.3. FN Molecular Conformation

**Figure 2:**
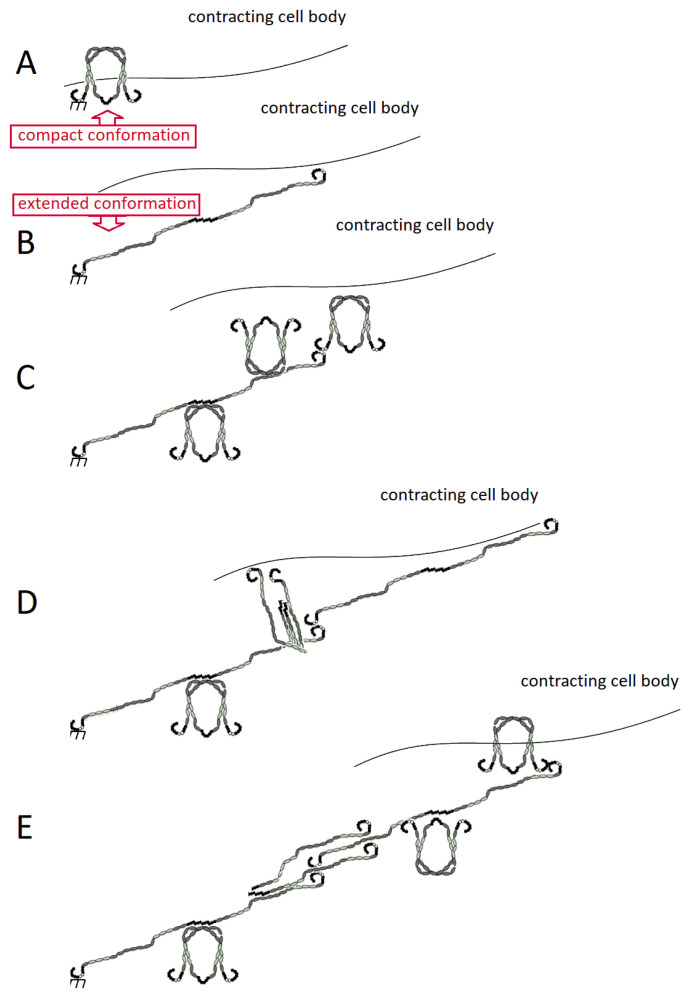
Assembly of FN fibrils from soluble FN. (**A**) FN exists in a soluble conformation, which binds to integrins on the cells surface. (**B**) Actomyosin force extends FN, facilitating (**C**) FN–FN binding. (**D**) As additional soluble FN molecules bind to the fibril, additional integrin binding drives progression and assembly of an insoluble fibril. (**E**) FN–FN interactions are detailed in the text, and involve interactions between homotypic Type I domain (black circle) interactions, and heterotypic Type I and Type III domain (gray oval) interactions.

**Figure 3:**
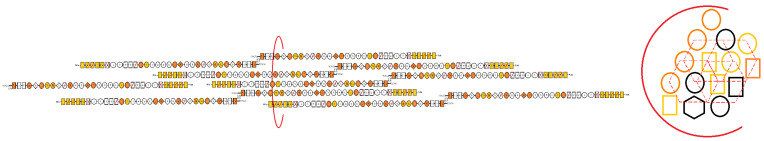
Assembly of FN fibrils. FN assembly is facilitated by interactions between domains I^1–5^ (yellow rectangles) and several Type III domains (various circles). FN is in an extended conformation in FN fibrils, and as such, most likely enables multiple FN–FN binding events within the cross section of the fibril. Since stretched III domains can bind via β-strand addition, it is possible that fibril maturation and insolubility is driven by these binding events. Computational models of FN fibrillogenesis have assumed a hexagonal packing organization [7,25], but actual molecular organization is not known. A cross section on the left demonstrates how different domains within the FN moelcule may interact with a range of domains from neighboring FN molecules within the fibril.

Although FN domains are connected end to end, FN adopts a compact conformation in which III^2–4^ of one subunit interacts with III^12–14^ of the other, folding the dimer upon itself [21]. Further evidence supports that I^1–5^ is necessary for FN to fold into this compact conformation, while domain deletion mutants confirm that III^1–3^ is nonessential [42]. This conformation is present in physiological conditions but is extended at high ionic strength or high pH [43]. After secretion, stabilized soluble FN binds to cell surface integrins, which cluster and reversibly bind to FN. Subsequent recruitment of integrins begin to unfold the conformation into a linearized structure, further stretching its domains into extended morphologies that allow additional soluble FN to be deposited in a continual, iterative process (Figure 2). The resulting structure would require interaction between different types of FN domains within the cross section of the fibril; a represenative schematic of how this might appear is shown in Figure 3. It is worth noting that this fibrillar structure is consistent with work that has shown that the N-termini (yellow rectangles in Figure 3) align in an anti-parallel configuration within fibrils [44,45].

## 3. Fibronectin: The Fibril

### 3.1. Assembly of FN Molecules into a Fibril

Soluble, molecular FN can be assembled into insoluble FN fibrils by cells in a process termed either FN fibrillogenesis or FN matrix assembly. This process is a self-initiated, aggregating process that requires cell-applied stretch (Figure 2). FN binds primarily to α${}_{5}$β${}_{1}$ and α${}_{v}$β${}_{3}$ integrins that are tethered to the actin cytoskeleton via focal adhesion complexes [1]; while α${}_{v}$β${}_{3}$ integrins can mediate FN fibril assembly in the absence of β${}_{1}$ integrin [46], FN fibril assembly is typically mediated by α${}_{5}$β${}_{1}$ integrins.

Myosin motors generate contractile forces that are applied to FN via the actin filament/focal adhesion/integrin axis which stretches open cryptic FN–FN binding domains. Unfolding of these domains allows for subsequent soluble FN binding via the 70 kDa N-terminus of the protein [13,25,47,48,49]. This early FN matrix becomes the primordial ECM and is the first ECM protein assembled by cells during wound healing and embryonic development [1,13,47].

While it is well-established that cell-derived contractile forces are necessary to expose cryptic binding sites in FN that facilitate FN fibrillogenesis [48], the location and number of these cryptic binding sites are less clear. Most evidence suggests that cryptic binding sites are within the Type III domains: III${}^{1}$ and III${}^{2}$ are considered the critical binding sites for fibrillogenesis, but other studies have shown that III^4–5^, III^7–10^ and III^12–14^ exhibit binding to the 70 kDa N-terminal fragment of FN [1], suggesting a potential role for these domains in FN fibrillogenesis. Though multiple domains in FN exhibit FN affinity, fibril assembly is specifically reliant on two regions: I^1–5^ and III^1–3^; mutants lacking these regions do not assemble FN matrices [50,51]. Furthermore, stabilizing III${}^{2}$ with a disulfide bond to prevent unfolding significantly depletes fibril formation [52], suggesting that this relationship is directional: the primary event in FN matrix assembly is the unfolding of Type III domains followed by subsequent binding of the I^1–5^ region of a soluble FN molecule [53]. The I^1–5^ region of soluble FN is capable of binding various proteins via β-strand addition [54], and steered molecular dynamics simulations have indicated that stretched Type III domains have stable intermediary conformations in which β-strands along the edges of the domain twist to align and become extended and exposed [20]. As such, it has been proposed that FN fibrillogenesis is facilitated through β-strand addition. Given the homologous structure of Type III domains, it has been suggested that all Type III domains may be capable of binding the I^1–5^ in a stretch-dependent manner [7,25]. This is especially interesting since both I^1–5^ and III^2–4^ play a role in holding FN into its soluble, compact conformation [22,42]; this would suggest a mechanism by which FN–FN binding is limited when FN is in a soluble, compact conformation but becomes more available once FN is stretched into an extended conformation.

### 3.2. Destruction and Turnover of FN Fibrils

FN fibrils are largely insoluble, and assembly is not a reversible process; however, several catalytic agents have been identified that cleave FN. Most identified sites are highly specific in their digestion of FN and are suspected to work only on unfolded regions of the protein. Proteolytic species including thermolysin, plasmin, thrombin, trypsin, cathepsin D and chymotrypsin all fragment FN into reproducible pieces (see Figure 1 for cleavage sites), but less clear is definitive segmentation using matrix metalloproteinases (MMPs). MMP activity on FN has been detected in 18 out of the 25 members of the MMP family: collagenases (MMP-1,-8,-13), gelatinases (MMP-2,-9), stromelysins (MMP-3,-10,-11), matrilysins (MMP-7,-26), membrane-types (MMP-14,-15,-16,-17,-24,-25) and others (MMP-12,-19). MMP-2 binds via its hemopexin-like (PEX) carboxyl-terminal domain, but the corresponding region on the FN molecule has yet to be fully elucidated. Membrane Type 1-MMP (MT1-MMP) has also been implicated in FN matrix turnover [55]. It has been shown that MMPs can remain in solution and continually cleave FN after certain chromatography techniques; however, even after removing these, FN interestingly has been reported to have autolytic functionality [56].

### 3.3. Inhibiting FN Fibrillogenesis

Given the predominant role of FN fibrils in wound healing, development, and diseases, we need tools to inhibit fibrillogenesis experimentally to investigate its effects on the progression of the processes. Because FN fibrils require cell-generated traction forces, FN assembly can be suppressed with the exogenous addition of: the Rho-kinase inhibitor, Y27632; the Rho inhibitor C3 transferase; or the myosin II inhibitor blebbistatin [47,48]; however, somewhat pardoxically, increased contractile forces can also inhibit FN fibrillogenesis: calyculin A, an upregulator of myosin II activity, inhibits FN fibril growth [22]. Given that specific regions of the FN molecule have self affinity and facilitate fibrillogenesis, assembly of FN fibrils can also be inhibited with exogenous soluble 29 or 70 kDa fragments [53] as well as fragments containing specific whole- or partial-Type III domains, including III^1–2^ [52], III^4–5^ [57] and III^12–14^ [58]. Another FN fibril inhibitor was discovered in the bacteria, *S. pyogenes*. The functional upstream domain (FUD) of the F1 adhesin protein is a 49 amino acid peptide that effectively blocks fibrillogenesis in vitro and in vivo by binding to the Type I domains in the N-terminal of the 70 kDa fragment. Although its effects are not as strong as Y27632, FUD significantly inhibits FN fibrillogenesis without affecting cell secretion of soluble FN, attachment of cells to a substrate, or cell spreading [47].

### 3.4. Artificially-Derived FN Fibers

Several attempts have been made to mimic FN assembly in cell-free assays, with variable likenesses to physiological fibrils (summarized in Table 1). The first technique consists of mixing a fragment of the FN III${}^{1}$ domain, termed ‘anastellin’, with monomeric FN at a ratio of 4:1, where three of the anastellin molecules have been shown to bind specifically to the III^1–3^ region of the FN monomer. The anastellin-III^1–3^ complex must contain at least three binding sites for other FN; one binding site would only generate dimers, and two sites would form linear chains. The complex architecture of these artificially derived fibrils suggests that at least 3 sites exist, which are presumably within unfolded Type III domains. Anastellin also binds to III${}^{11}$, and both bindings cause conformational changes that expose cryptic thermolysin-sensitive sites [59]. Anastellin-FN-coated surfaces have greater cell adhesion, and the anastellin-FN aggregates readily form fibrillar structures that resemble cell-derived FN fibrils at the light microscope level; these fibrils have been dubbed ‘superfibronectin’ (sFN), as they assemble in the absence of cell-applied force [59]. Some studies have suggested that the formation of sFN is similar to the mechanism of assembly for cell-derived FN fibrils and that understanding its polymerization may reveal intricacies into the unfolding-refolding aspects of FN assembly. Interestingly, sFN assembly does not require FN dimerization nor the N-terminal of the 70 kDa fragment, both of which are critical for cell-derived FN matrix assembly. Anastellin also possesses exposed β-sheet edges that are susceptible to amyloid formation. These behaviors are specific to anastellin; other truncated FN Type III domains do not demonstrate similar behaviors. Furthermore, it is unlikely that anastellin exists in vivo, and as such, would not play a role in cell-derived FN fibril formation. Interestingly, at high concentrations (40 μM), anastellin blocks FN matrix formation, causes loss of the EDA epitope, and dissolves established matrices in 16 hours. High concentration anastellin injections have thwarted angiogenesis and tumor growth, suggesting that anastellin may serve as a competitive inhibitor to cell-derived FN fibril assembly at high concentration [60].

The second technique for generating cell-free derived FN fibers is to manually draw them from purified, high concentration pFN droplets. These artificially derived FN fibers rely on shear forces at the air/liquid interface to stretch FN molecules and have been reported to resemble cell-derived FN fibrils. Depending on the concentration of soluble FN, artificially derived fibers have more homogeneous diameters, with average sizes between 2 and 5 µm, and can be manually drawn in any desired pattern, with fibril lengths of 1 to 2 cm. These may be dried out in their preparation and are often draped across microfabricated trenches or onto pre-strained or stretchable sheets [63,64,66]. However, they differ considerably from cell-derived fibrils in their diameter, organization, and mechanism of assembly. For example, artificially derived fibers can be stretched more than 8-fold whereas cell-derived fibrils can be maximally stretched 4-fold their resting length. As such, these fibrils may not fully recapitulate the mechanics of cell-derived FN fibrillogenesis. Despite these differences, both cell-derived and artificial fibers have reported to be extremely elastic and recoil after relief of mechanical stress [61,63,64]. Although artificial FN fibers have been described as collections of bundles of cables similar to configurations of FN fibers as confirmed by cryo-scanning electron microscopy, they undoubtedly lack several features of cell-derived fibrils. Physiological FN fibers are proposed to be held together by hydrogen bonds, intermolecular β-strand swapping, disulfide bonds, and weak electrostatic interactions and are assembled in a cell-force dependent manner. They are also interwoven with other ECM proteins and decorated with small molecules, which have been suggested to create unique fibril mechanics. Cell-free assembled fibers almost certainly lack these bonds and cannot completely replicate FN fiber behavior as a result of cell-derived fibrillogenesis given the complexity of lateral interaction between adjacent FN molecules.

### 3.5. Models of FN Fibrillogenesis

To better ascertain the potential mechanism of FN fibrillogenesis, computational models have been constructed to predict fibril morphology and mechanics from first-order principles of stretch-driven domain opening, binding, and assembly (summarized in Table 1). These computational models are able to predict length:thickness ratios, stretched:relaxed length ratios, and force-stretch relationships consistent with experimentally observed fibrils [7,25]. Simulations suggest that a single FN–FN binding site is not sufficient for FN fibrilogenesis, but instead requires multiple binding sites, with at least one binding site in the N-terminal of III${}^{10}$ [25]. This same model suggests that two distinct populations of fibrils exist: stably stretched fibrils (SSFs) which gradually stretch in time before reaching a threshold and are stably attached to their substrate; and fluctuating stretched fibrils (FSFs) which fluctuate around an average value attributed to the stochastic formation and breaking of bonds between integrins and the FN fibril [25]. SSFs are predicted to be larger with more FN molecules, while FSFs are smaller. The existence of these two subtypes has not been confirmed experimentally but could have profound impact on the way in which a cell senses its surroundings via FN fibrils, particularly considering the heterogeneous population of fibril morphologies that has been observed experimentally.

Another system proposed to predict the high extensibility and nonlinear stiffness of FN fibrils is the microstructural modeling approach which compares experimental molecular data to fiber length scales in attempts to explain how dynamic molecular conformational changes and intermolecular connections develop bulk FN fibril characteristics and overall ECM properties [67]. Utilizing the worm-like chain interpolation and Bells’ unfolding probability to model networks of molecules as springs connected at nodes, this method relies solely on entropic polymer elasticity and unfolding FN Type III domains, coupled with parameters obtained through spectroscopies, to simulate stretching at pulling speeds (0.91 um/s) slower than atomistic modeling. The network configuration is found by minimizing the strain energy with respect to node position, but the system details fibrils with cross sections of 1–2 molecules such that a molecular concentration conversion provided by deep UV transmission experiments is necessary to scale up to micrometer sized FN fibrils, along with certain specific assumptions about their end-to-end configuration and length of individual molecules. It displays its most predictive power in the early stages of deformation with no unfolded domains and in reinforced configurations where a single FN molecule is paralleled with two in series but less so for simple chains of FN molecules strung end to end. The model adds to the hierarchal arrangement of FN fibril cross sections in vitro, that is, molecularly, they are likely overlapping and contain prestressed Type III domains. However, it ascribes FN fibril bulk properties to bundled FN nanofibers and requires experimental input parameters, which interestingly have opposing force behaviors with respect to end-to-end size compared to in silico predictions; it was suggested this may be due to quaternary structure extension over domain deformation, limiting its appropriateness to early fibrillogenesis.

A mesoscopic model has also been developed that addresses some of the limitations of the aforementioned since they neglect refolding events and interchain play [65]. The mesoscopic model incorporates cryptic binding sites to facilitate fibrillogenesis by describing fibrils as composed of molecules of seven membered hexagonal springs, some of whose internal bonds may be broken into an extended conformation. The Z backbone is fixed due to the Lennard-Jones potential and arranges linear molecular chains into bundled fibrils held together by adjacent interactions. It calculates force from domain velocity via hybrid Brownian dynamics-Monte Carlo simulation, following a conventional overdamped Langevin equation, and interchain rupturing and reformation are governed under Bell conditions. The model reveals that yielding and rupture are hindered by cryptic sites that act to strengthen lateral chain connections and give fibrils their nonlinearity and strain hardening behaviors. Furthermore, it predicts two strain relaxation periods that have been experimentally confirmed at a physiologically observed 400% stretch: short time-scale resettling of stretched bonds, then a second period of domain refolding and reorganization. However, the model does not differentiate between types of cryptic sites: not only do certain Type III domains contain hidden cysteine residues, unfolding can yield extended β-strands that may exhibit nonspecific binding as well as hydrophobic residues that may also display FN affinity; similarly, it is nonselective in the unraveling of domains such that their site-specific strain thresholds, opening sequences, and functional activations are lost. Notably, this system may be applied to other multidomain proteins and complexes by augmenting number, type, and strength of bonds.

## 4. Interactions of FN with the Extracellular Matrix

FN interacts with many other ECM proteins as well as small molecules, growth factors, glycosaminoglycans (GAGs), cell surface receptors and other FN molecules [1,19,68]. These connections provide key mechanical and chemical signals to induce specific cell behaviors including differentiation and epithelial-mesenchymal transition (EMT) [69], and their misregulation promotes scarring, tumorigenesis, fibrosis, and developmental defects [70,71]. Given the vast affinity FN has for the multitude of different species within the ECM (reviewed in [72,73]), here we highlight specific interactions that may play important roles in mechanochemical signaling of FN fibrils:

Collagen and Collagen-Modifying Proteins. As the primary component of assembled tissue, the role of collagen assembly in mechanobiology is substantial. FN domains I^6–9^ and II^1–2^ bind collagen-1, and more effectively gelatin, serving to sequester denatured collagenous debris [74]. FN fibrillogenesis and collagen fibrillogenesis have a complex relationship [75,76], with evidence showing a role of FN in regulating collagen assembly [77,78] and a role for collagen in regulating FN assembly [79,80]. In addition to these direct interactions between FN and collagen assembly, other proteins play a role in modulating FN/collagen interactions. Thrombospondin-2 contains domains that have affinity for FN and help modulate ECM assembly and remodeling [81]. Periostin bears a secretory signal peptide and has shown to aid FN in secretion, localizing within the endoplasmic reticulum of fibroblastic cells while aiding as a scaffold between collagen fibers [82]. The 30 kDa active form of the collagen-cross-linking enzyme LOX has been shown to have great affinity for cFN but not pFN, which may explain the necessity for an established FN matrix prior to collagen fibril maturation or elastin crosslinking [83].

Tenascin-C. Tenascin -C is an ECM protein that is often upregulated in solid tumors [84]. Although it seems to have evolved before FN, tenascin-C has several FN Type III-like domains which may colocalize with other FN Type III domains or be closely associated with FN matrix as it binds directly to FN [85], as well as to collagen and perlecan through its FN III^3–5^-like domains [84]. Several of the FNIII repeats possess the RGD motif in the same exposed looped conformation as in FN, such that it may have primarily served as an integrin ligand. Similar to FN, tenascin-C displays distinct post-translational modifications, affinities, and proteolytic susceptibility [84].

Fibrin Clots. Both an N- and C-terminus binding site exist for fibrin in FN domains I${}^{4,5}$ and I^10–12^, respectively. These binding sites are thought to play a role in cell adhesion and migration into fibrin clots as well as in facilitating macrophage clearance. Factor XIIIa, a plasma transglutaminase, crosslinks fibrin polymers, and its FN binding site is conveniently located at the N-terminal, just before domain I${}^{1}$ [86].

Fibrillin and Associated Family Members. Microfibrils form a major structural component of the ECM, and misregulation of microfibril assembly is implicated in disease states including Marfan’s Syndrome. FN is essential for microfibril formation, including fibrillin; siRNA knockdown experiments of FN indicate significantly impaired microfibril formation in the absence of FN expression [87]. FN affinity for fibrillin may be inhibited by gelatin, suggesting its binding site lies between FNII${}^{1}$ and FNI${}^{9}$ [88].

Growth Factors. Members of the transforming growth factor β (TGF-β) family, fibroblast growth factor (FGF) family, platelet-derived growth factor (PDGF) family, hepatocyte growth factor (HGF) family, and vascular endothelial growth factor (VEGF) family all have multiple binding locations within FN, but most seem to share affinity for FN III^12–14^ [68,89]. Furthermore, latent TGF-βbinding proteins (LTBPs) colocalize with FN fibrils, which may increase the capacity of the ECM small molecule reservoir [90]. Localization of growth factors to FN occurs without disrupting the binding of these cytokines to their corresponding receptor [68] and thus may serve as a mechanism to upregulate signaling; for example, localization of TGF-β1 to FN fibrils upregulates TGF-β1-induced EMT [69].

Glycosaminoglycans and Proteoglycans. The glycosaminoglycan (GAG) heparin, along with proteoglycans (PGs) containing heparan sulfate or chondroitin sulfate side chains exhibit FN affinity.These include members of the syndecan family, which enhance cell-FN interactions with integrins [6,91]. Interestingly, perlecan-FN substrates have been shown to have anti-adhesive effects during cell attachment but do not display similar effects on perlecan-laminin substrates. Heparin and heparan sulfate, but not hyaluronan or chondroitin sulfate, also reduce adhesion to FN, the effects of which can be diminished with heparinase treatment. These GAG–PG interactions form the hydrogel structure of the ECM, further supported by FN, collagen and elastin networks [92].

Bacterial Wall Proteins. Many bacteria express cell wall-anchored FN binding proteins that align antiparallel with the 29 kDa fragment to form a tandem β-zipper. These include *S. aureus*, *S. dysgalactiae* and *B. burgdorferi*. Cell wall proteins from *S. pyogenes* and *S. equismilis* also bind to this region as well as to the collagen binding fragment. Several of these bacterial wall proteins have been shown to inhibit FN fibrilogenesis (discussed above) [93,94,95,96].

Integrins. The RGD site located in III${}^{10}$ is known to bind α${}_{v}$β${}_{6}$, α${}_{v}$β${}_{3}$ and α${}_{v}$β${}_{8}$ integrins [11], while both the RGD site in III${}^{10}$ and the PHSRN synergy site located in III${}^{9}$ [97] binds α${}_{5}$β${}_{1}$ and α${}_{IIb}$β${}_{3}$, but not α${}_{v}$-containing integrins [98]; similarly, sites common to alternative splicing often contain LDV, REDV and EDGIHEL and thus affinity for α${}_{4}$β${}_{1}$, α${}_{4}$β${}_{7}$ or α${}_{9}$β${}_{1}$ integrins [13]. α${}_{5}$β${}_{1}$-integrin-mediated cell adhesion has also been demonstrated to interact with N-terminal fragments containing repeats I^1–9^ and II${}^{1,2}$ (reviewed in [13]). Integrin binding is affected by the degree of stretch applied to the fibril: when the RGD sequence in III${}^{10}$ is confined such that it stays in close proximity to the PHSRN site in III${}^{9}$, FN binds both α${}_{5}$β${}_{1}$ and α${}_{v}$β${}_{3}$ integrins; when the PHSRN sequence is separated from RGD, FN preferentially binds α${}_{v}$β${}_{3}$ integrins [99].

## 5. FN Fibril Biophysics

FN fibrils serve as a critical link between cells and their surrounding, particularly in fibrotic disease states. As such, the mechanochemical signaling properties of FN fibrils are crucial to understanding cellular mechanoresponses. In the following section, we explore how FN fibrils may contribute to cellular mechanotransduction.

How do cells sense their environment? Mechanotransduction is the process of transmission of extracellular mechanical signals to the cell, which then cause the cell to modulate biochemical responses. These mechanical signals are transmitted via integrins and other membrane-bound receptors, which are in turn linked to the cytoskeleton. Mechanotranduction allows for cells to respond to haptotactic (adhesion), rheotactic (fluid flow), curvotactic (cell length-scale curvature), topotactic (topographical), durotactic (stiffness), mechanotactic (mechanical stress), and viscotactic (viscosity) signals [100,101,102,103]. Studies in mechanotransduction have indicated that cellular response to mechanical signals include altered cell migration, differentiation, transcriptional activity, proliferation, morphology, and apoptosis (reviewed in [104]).

How does FN’s unique structure elicit cellular function? The ECM plays a critical role in mechanotransduction signaling as it provides critical cues to guide cell fate, morphology, movement, remodeling and differentiation [2]. FN fibrils have the potential to alter mechanotransduction signals in two significant ways: (1) FN fibrils serve as a mechanical link between cells and the surrounding ECM. Given that cells are mechanically sensitive and responsive to their environment, it stands to reason that mechanotransduction from the ECM to cells depends on the specific biomechanical properties of FN fibrils since FN plays a pivotal role in the developing ECM; (2) FN fibrils are a bioactive molecular reservoir. As discussed above, FN fibrils bind a wide array of ECM molecules, growth factors, and other small molecules that are essential for and influence further ECM assembly and remodeling. The state of FN fibril stretch affects their binding affinity and may reveal cryptic binding domains, in turn binding other proteins that can affect the mechanical properties of FN fibrils; for example, collagen-1 preferentially colocalizes with relaxed FN fibers, shielding them from cell traction forces [105]. Therefore, the mechanical behavior of FN fibrils may lead to temporal or cyclical binding events in the ECM in a force dependent manner [106].

What level of predictive power do alternative FN fibril strategies offer towards cell-derived FN biomechanics? Computational models and in vitro artificial FN fibers have given insight into fibril mechanics, and while these demonstrate fundamental characteristics of force- and stretch-dependence in FN assembly, they fail to accurately report certain aspects of the biophysics of cell-derived fibrils. Computational simulations of FN fibrils show length-dependent, viscoelastic loading behaviors and stretch-mediated exposure of a cryptic binding site for assembly, but differences over predicted extensibility, mechanical strength and binding kinetics create a demand for cell-based studies [25,65]. Furthermore, the prediction of sub-type populations of FN fibrils [25] has complicated the ability of models to predict the mechanical behavior of hundreds of interconnected FN molecules and challenges the current understanding of FN mechanics. Analysis of artificial fibers have demonstrated 8-fold extensibility, hysteresis, viscoelasticity, creep with time dependent recovery and selective reversibility, nonlinear stiffness, plastic deformation and force-generated fibrillogenesis [63,64,66,106]; however, only 4-fold extensibilities have been observed in cell-derived fibrils [59], and ambiguities remain between cell-derived fibrils’ reversibility and plastic performance, with some experiments showing the physical destruction of certain cell-binding sites when stretched.

How much force is needed to generate cell-derived FN fibrils? Experiments have revealed that a force range of 2–5 nN facilitates fibrillogenesis [22,47]; however, many components go into the application of force to FN fibrils, and the lack of understanding of both the mechanism of FN elasticity and FN assembly complicate the question. In terms of force applied to the fibril, integrins have been shown to form noncovalent catch bonds whose lifetime increases until a maximal force between 10 and 30 pN is reached. The focal adhesion protein vinculin is under a constitutive 1–2 pN load by the cytoskeleton, and the focal adhesion protein talin unfolds between 5 and 25 pN [107,108] These may amass to cell contractile forces up to 100 nN per focal adhesion, which has been shown to strain computational FN nanofibers [106].

What role do mechanics play within individual FN subunits? In terms of FN domain unfolding, atomic force microscopy (AFM) studies have indicated that forces on the order of 100 pN are needed to unfold Type III domains; however, these experiments were performed on individual domains that were stretched at pulling rates much higher than what an FN domain would experience in vivo, considering cells assemble fibrils with near zero pulling speeds. Conversely, a subpopulation of Type III domains have been shown to spontaneously unfold and refold in the absence of applied force, with FN having folding kinetics ranging from 4 × 10${}^{-3}$ s${}^{-1}$ for III${}^{1–2}$ to 2 × 10${}^{-2}$ s${}^{-1}$ for III${}^{10}$ and III${}^{13}$ [59]. In this situation of spontaneous unfolding, the key parameter would be the force needed to keep a Type III domain from refolding, not the force needed to unfold.

How do FN molecular mechanics contribute to overall fibrillar extensibility? Another key aspect of forces applied to FN fibrils is the mechanism by which FN fibrils stretch. Experimental evidence have demonstrated that cell-derived fibrils can be stretched up to four times their resting length [61], which has implication on domain unfolding and subsequent ECM remodeling. Two different mechanisms have been proposed for the mechanism of FN stretch: one in which elasticity is attributed to Type III domains unfolding [109], and a second that contends that FN molecules extend from a compact conformation to an extended conformation within fibrils [110]. Experimental evidence indicates that 9 of the 15 Type III domains in FN do not unfold, and wormlike-chain models of domain unfolding suggest that the magnitude of cell-derived forces is insufficient to generate the 4-fold stretch observed in cell-derived fibrils [21]. In contrast, computational models of FN assembly that utilize only Type III domain unfolding as a mechanism of FN fibril stretch are able to replicate the FN extensibility seen in vitro, including Type III domain opening observed experimentally [7]. Furthermore, the extensibility of FN fibrils must be accommodated through the breaking of bonds that stabilize the protein structure of the individual FN molecules; this bond breaking could also affect the entire fibril structure, leading to plastic deformation [106], which could have profound implications on the mechanical properties of FN fibrils over time. Older fibrils, which have been repeatedly stretched by cells, may have profoundly different mechanical properties than newly assembled fibrils. Interestingly, stretch-dependent glutathionylation of FN was recently show to irreversibly alter the mechanical properties and binding affinities of FN, effectively signaling downstream cascading events [111]. This may be involved in oxidative stress-related development of pathologies, proving that time and stimulant dependent changes in the ECM affect its structure-function relationship.

Taken together, these studies suggest that cellular components are capable of producing a recruitment mechanism in order to cluster nascent FN molecules into robust fibrils capable of withstanding large forces and deformations which may then be stretched to reveal cryptic binding domains for further assembly; however, there is no cohesive consensus on the mechanism of fibrillogenesis. Whereas the pioneering work done by Kron and Spudich in developing the myosin motility assay definitively details the minimum components needed for actin contractility and quantified the dynamics of a major cytoskeletal protein [112], there are no minimal models for FN fibrillogenesis that incorporate only minimally necessary components and match computational and in vivo responses, and certainly none that encompass the relation between the cytoskeleton, focal adhesion, and FN. While a minimal model of FN assembly is undoubtedly more complex than minimal models for actin polymerization or myosin motility, pursuit of such a system would dramatically advance the field. It has been demonstrated that myosin driven, actin-based contractile forces, coupled to integrins through focal adhesion proteins, facilitate attachment and generate contractile forces to assemble FN molecules in fibrils with morphologies indicative of their environment; however, we have yet to quantify: the ATP energy production requirements for FN fibril assembly by myosin motors; the level of actin polymerization needed to support cellular contraction necessary for fibrillogenesis; the necessity and discrepancy between specific integrin populations in the process; definitive focal adhesion proteins in the process; the total cellular force generation with respect to substrate and biochemical conditioning that could be utilized for fibril formation; the minimal forces mandatory to deform FN molecules from compact conformation into extended, stretched or clustered forms to facilitate fibrillogenesis; or how specific mechanical environmental cues direct or deform fibril formation or contribute to production level to maintain a desired homeostasis. Cell-derived FN fibrils themselves have yet to be fully mechanically profiled: they are elastic, have demonstrated viscoelastic parameters, can be unfolded and domains can be opened, have high stretch ratios and have the potential to be mechanically differentiated through their hierarchical arrangement in cross section and variability in length, but we have not characterized specific moduli, rupture mechanics, thermal or temporal components, forces for fibrillogenesis, the mechanism attributing to their extensibility, or how to subcategorize FN fibrils based on physical and mechanical properties. As much as we know biochemically about FN, we know considerably less about its mechanical value as a material.

## 6. Strategies for Studying FN Biophysical Properties

Given the prominent role of FN fibrils in cellular mechanotransduction, we need approaches to study these mechanics. The gold standard for quantifying FN matrix assembly is the deoxycholate detergent (DOC) insoluble electrophoresis assay originally defined in 1983 [113]. In this assay, assembled FN fibrils are extracted from surfaces and quantified by Western blot following resolublization in DOC. This is traditionally coupled with immunofluorescence microscopy, where assembled fibrils can be visualized as rope-like structures. These assays, while quantifying the degree of assembly, give no insight into fibril mechanics. To address this, mechanobiological assays have been developed to measure the material properties of the ECM and its constituents.

On a whole-tissue level, biaxial tensile testing on excised specimens can be used to quantify overall ECM mechanical properties, and these properties can be correlated with ECM composition, ECM fiber alignment, and ECM morphology [114,115]. However, these assays fail to specifically probe the contribution of FN fibrils. Another method has used microcontact printed FN on a PIPAAm substrate that when released, form ‘nano fabrics’ or ‘FN textiles’ [116]. These create a web of FN fibrils, but, similar to artificially derived FN fibers, may not capture in vivo FN fibril properties. On a mesocellular level, microcontact printing, microfluidics and microfabrication have been combined to study the role of FN fibrils in transmitting forces, using either cell-derived fibrils [22,47] or artificially derived fibers [64,109], but these studies do not specifically investigate FN fibril mechanics. In the interest of measuring cell-substrate forces, some techniques such as AFM [117], (astigmatic) traction force microscopy [111], elastic resonator interference stress microscopy [118], or hex dot microcontact printing [119] tend not to focus directly on ECM response to deformation, where often FN, if involved, may only be considered for facilitating substrate attachment or is subjected to the survey only at the molecular or domain constitution [110].

Several assays have been developed to specifically probe into the stretching of FN fibrils. In one assay, FN fibril stretch is quantified via Forster Resonance Energy Transfer (FRET): a FRET acceptor fluorophore is used to label native free cysteines, while a FRET donor fluorophore is randomly added to FN. As FN fibrils are stretched, the FRET efficiency changes, resulting in quantification of fibril elongation [64,109] This assay has shown the contribution of strain-induced Type III domain unfolding to overall fibril extensibility. It allows for mechanical stress application to be controlled externally and measured optically as well as certain domain ranges’ force characteristics according to fibril molecular concentration. This technique has been demonstrated to show distinct differences in the state of stress between manually drawn artificial fibers and cell-derived FN fibrils. Careful consideration must be employed during the fluorescent dye labeling since end-to-end configuration and random amine labeling can lead to signal overload, which may dilute force values obtained since its force calculation is specifically distance determined [63]. Another approach to quantify FN stretch is the use of thiol-reactive dyes to label buried cysteines [21]. This approach has been used to determine the unfolding of FN Type III domains; results from these studies have identified Type III domains that unfold during FN fibril stretch as discussed above. While this assay provides a measure of domain unfolding, it is primarily binary: it only indicates whether domains unfold, and not a quantifiable displacement of the domains.

At the molecular level, AFM has been used to quantify the mechanics of individual Type III domains [24]. These studies give the most specific insight into FN mechanics to date; however, as discussed above, the macromolecular structure of FN fibrils is complicated, and thus, the mechanics of individual FN domains may have little correlation with whole-fibril mechanics. However, these data are still important: they have been used in computational models of FN fibrils discussed above, and have been used to predict whole-fibril morphologies.

## 7. Commentary and Outlook

In this review, we have discussed the structure and function of FN fibrils at both the molecular and fibrillar levels. Given that these fibrils occupy a critical location between cell and surrounding tissue during wound healing, development, and in many disease states, it is of great importance to understand both the mechanism of assembly and the resulting mechanical properties of FN fibrils.

While extensive work has been done to improve our understanding of FN fibril assembly and mechanics, several areas remain to be elucidated. While computationally predicted FN fibrils and artificially derived FN fibers have given insight into fibril anatomy, the discrepancies between these fibers and cell-derived fibrils limit the impact of these studies. Furthermore, the complicated structure and interwoven nature of FN fibrils in the ECM impairs the ability to ascertain individual fibril mechanics, growth, and signaling.

A key missing experimental tool is a method to probe the mechanical properties and assembly of isolate, cell-derived FN fibrils. Such a system would allow us to answer several unanswered questions including: what are the mechanical properties of individual FN fibrils, and how does this affect cellular mechanosensation? Are mechanical properties of FN fibrils changed based on binding of other ECM proteins, cross-linker proteins, and/or tethered growth factors? Are the mechanical properties of FN fibrils changed by repeated stretching over time, such that FN fibrils exhibit a “memory” of applied forces? Does the signaling and/or structure of FN-bound proteins change relative to freely diffusing proteins? These questions remain as interesting avenues of study into the important role of FN fibril mechanics. 

## Figures and Tables

**Table 1:** Properties of Experimentally Observed and Computationally Predicted FN fibrils. Both cell-derived and artificially derived FN fibrils have been studied in vitro, but there are observable differences between the two. Computational models of both cell-derived and artificial fibrils have been developed, with distinct approaches and assumptions.

	Cell-Derived	Artificially-Derived
**Experimental**	• 5–1000 nm, heterogeneous diameters [44]	• 2–5 µm, homogeneous diameters
	• <5 to >50 µm length [61]	• 1–2 cm length [63]
	• 3–4-fold stretch [61]	• 5–8-fold stretch [63,64]
	• preliminary mechanical data available for elastic, viscoelastic, and cyclical properties (unpublished)	• high, reversible strain; low rupture events [63]
	• formed by cell secretion and stretch via self affinity [24,48]	• formed from surface tension/air-liquid interface [63,64]
	• isoforms determined by soluble content and alternative splicing	• millions of FN molecules, isoforms determined by solution preparation [63]
	• insoluble, typically remain submerged in aqeuous environment [62]	• insoluble, may be dried in preparation [63]
**Computational**	• 10–50 nm, hexagonally packed cross-section with randomized orientation depending on FN molecule spring configuration [7]	• density considered over cross-section, often organized as small clusters of linearized chains with cylindrical geometries [65]
	• 1000–2000 nm lengths [7,25]	• lengths measured by bond stretch, limited by force laws [65]
	• 2–3-fold stretch [7,25]	• stretch set by stiffness parameter and force applied [65]
	• distinct subtype populations predicted [25]	• demonstrates stress relaxation with domain extension with destabilized drops, dependent on number of neighboring bonds [65]
	• modeled as a different number of springs in series with unique stiffness values [7,25]	• designed as series of domain repeats [65]
	• hundreds of molecules, isoforms set as combinations of springs [7,25]	• less than 100 molecules [65]
	• n number of integrin clutch states with reversible binding [7,25]	• random FN–FN domain interaction within the fibril during stretch [65]

## Data Availability

Not applicable.
